# Comments on previous psychological *Tai-Chi* models: *Jun-zi* self-cultivation model

**DOI:** 10.3389/fpsyg.2022.871274

**Published:** 2022-09-15

**Authors:** Jin Xu, Nam-Sat Chang, Ya-Fen Hsu, Yung-Jong Shiah

**Affiliations:** ^1^Department of Psychology, School of Education Science, Minnan Normal University, Zhangzhou, China; ^2^Graduate Institute of Counseling Psychology and Rehabilitation Counseling, National Kaohsiung Normal University, Kaohsiung, Taiwan; ^3^Hui Lan College, National Dong Hwa University, Shoufeng Township, Taiwan

**Keywords:** self, *I-Ching*, *xiao-ren*, *jun-zi*, *Jun-zi* self-cultivation model, Tai-Chi

## Abstract

In this article we describe four previous *Tai-Chi* models based on the *I-Ching (Book of Changes)* and their limitations. The *I-Ching*, the most important ancient source of information on traditional Chinese culture and cosmology, provides the metaphysical foundation for this culture, especially Confucian ethics and Taoist morality. To overcome the limitations of the four previous *Tai-Chi* models, we transform *I-Ching* cultural system into a psychological theory by applying the cultural system approach. Specifically, we propose the *Jun-zi* (君子) Self-Cultivation Model (JSM), which argues that an individual (小人, *xiao-ren*) can become an ideal person, or *jun-zi*, through the process of self-cultivation, leading to good fortune and the avoidance of disasters (趨吉避凶, *qu-ji bi-xiong*). The state of *jun-zi* is that of the well-functioning self, characterized by achieving one’s full potential and an authentic, durable sense of wellbeing. In addition, we compare egoism (*xiao-ren*) and *jun-zi* as modes of psychological functioning. The JSM can be used to as a framework to explain social behavior, improve mental health, and develop culturally sensitive psychotherapies in Confucian culture. Finally, an examination of possible theoretical directions, clinical applications, and future research is provided.

*Heaven’s rule of change is that Tao (Tai-Chi) generates one, then one generates two forms (Yin and Yang). Then the two generate the three. The three generate everything. Everything in the universe follows the law of Yin and Yang. They are opposite forces but paradoxically they also are complementary, interconnected, and interdependent in the universe and they give rise to the others as they interact with the others* (*Daodejing*, Chapter 42).

## A brief history of the *I-Ching* (*Book of Changes)*

With these words, Lao-Tzu (about 571–471 B.C.), the greatest Taoist philosopher and author of the *Daodejing* (道德經), echoes in principle a comprehensive statement about what is the ideal self state derived from the *I-Ching* (Anagarika, 1981; Cleary, 1986; Chia and Huang, 2005; Shiah, 2021). Taoism is grounded on the premise that one’s self and Heaven come from the same source, *Tao*, which refers to the universal law of everything in the world (Cleary, 1986, 1993; Xu et al., 2019) and manifests as the continuous alternation between the operations of *Yin* and *Yang* (一陰一陽之謂道) (*I-Ching*, The Great Appendix I, Chapter 24). The *I-Ching (Book of Changes)* describes Heaven’s rule of change, the most fundamental wisdom in Taoism (Xu et al., 2019).

The *I-Ching* was written by three important authors in different periods of Chinese history. It is said that in ancient times a horse covered with graphics and a turtle carved with characters on its shell were found in the Yellow River. The first edition of the book, which describes the first, primordial eight trigrams (先天八卦, *xian-tian ba-gua*), was allegedly written by Fu-Xi (伏羲) ca. 2800 to 2737 B.C. It is claimed that the last eight trigrams (後天八卦) were created by King Wen of the Zhou dynasty (周文王), who ruled China from 1112 to 1050 B.C. and extended the eight trigrams to 64 pairwise permutations of trigrams. It is said that Confucius, who lived from 551 to 479 B.C., added 10 annotated supplements (十翼) and a series of commentaries on the *I-Ching*. These works transformed the *I-Ching* from a divinatory system into a classic of Confucian and Taoist culture, cosmology, ethics, and morality. Each of the 64 pairwise permutations of trigrams represents a situation or state of affairs that an individual may encounter in his/her lifetime. The “famous teaching words,” which one consults to interpret the divinatory trigrams written by Confucius and his disciples, always advise that the best way to attain good fortune and to avoid misfortune (趨吉避凶) is to try one’s best to become a *jun-zi* (君子) and not a *xiao-ren* (小人). The teachings were habitually practiced by Confucianists and Taoists for self-cultivation. The cosmology described in this most important ancient Chinese book provides not only the metaphysical foundation of traditional Chinese culture, but also the basis for later Chinese medicine, *I-Ching* divination, *Feng Shui*, and Chinese astrology.

## Introduction

In order to initiate a scientific revolution against Western mainstream psychological theories constructed on the basis of Western, educated, industrialized, rich, and democratic (WEIRD) values (Joseph et al., 2010), Hwang (2019, 2023) proposed an epistemological strategy for constructing culture-inclusive theories with the aim to transform a cultural system into a set of psychological theories. Following the principle of cultural psychology “one mind, many mentalities” (Shweder et al., 1998, p. 871), Hwang’s (2015) cultural system approach argues that the deep structures and functions of human beings are the same across all cultures, but the mentalities developed in different ways in accordance with their respective cultures for the sake of adjustment to one’s lifeworld. His epistemological strategy includes two successive steps (Hwang, 2019). The first step is to develop formal theoretical models on self and social interaction. The second step is to use these formal models to analyze a given cultural system.

Because the *I-Ching* is the metaphysical foundation of the two major cultural heritages in China, Confucianism and Taoism, our main purpose in this article is to use Hwang’s epistemological strategy to construct the *Jun-zi* Self-Cultivation Model (JSM), based on the core wisdom of the *I-Ching*. Because the *I-Ching* and *Daodejing* are the earliest and most important classics in China. They have been interpreted and re-interpreted by numerous later philosophers, whose works can be conceptualized as examples of morphogenesis derived from the *Daodejing* and *I-Ching*.

## Previous *Tai-Chi*-related models and their limitations

There are currently four *Tai-Chi*-related models. The Wang team developed two of these models, which are similar to each other. The first of these is the *Tai-Chi* Model of the Confucian Self (Wang et al., 2019); the second is the *Tai-Chi* Model of the Taoist self and Buddhist self (Wang and Wang, 2020). In the first model, *Yin* represents the “small self,” the small, narrow, and dark self that serves the interests of the minority (in group). *Yang* represents the “large self,” the big, broad, and bright self that serves the interests of the majority (out group) (Wang et al., 2019).

The second Wang model has two sub-models. The first sub-model is the *Tai-Chi* Model of Taoist Self. *Yin* represents the “soft self,” which Wang et al. (2019) define as having the traits of self-reflection on “softness,” weakness, emptiness, simplicity, non-doing, and nature. *Yang* represents the “hard self,” which they define as having the traits of self-reflection on “hardness,” fullness, complexity, action, and artificiality. In the second sub-model, the *Tai-Chi* Model of Buddhist Self, *Yin* represents the “dusty self,” which they characterize as self-clinging to the five root annoyances or poisons (*kleshas*): self-ignorance (*avidya*), self-attachment (*raga*), self-aversion (*dvesha*), self-pride (*mâna*), and doubting Buddhist wisdom. *Yang* also introduces the “pure self,” the non-self (anatman or nir-atman) or the non-polluted and untroubled nature of reality (Wang and Wang, 2020).

The third *Tai-Chi* model is the Virtue Existential Career Model (VECM), developed by Liu et al. (2016). Based on the *Yin-Yang* concept, their secular counseling goal is to help people have a life based on the wisdom of the *I-Ching*. *Yin* represents receptivity and *Yang* represents creativity. Though *Yin* and *Yang* are considered as the opposite ends of a continuum, they fluctuate in terms of mutual completion and enhancement, generation by opposition, and joint production.

Fourthly, the initial *I-Ching* framework was developed and written in Chinese and is known as the Inward Multilayer-Stereo Mandala Model of the Unity of the Self and Heaven (IMMUSH) (Xu et al., 2019). This four-level model was fully based on *I-Ching* in a systematical manner adopting the Hwang’s epistemological strategy. But this model fails to explain how *Yin* and *Yang*, the well-known *Tai-Chi* symbols, are transformed into the unity of the self and Heaven, that is equivalent to *Tai-Chi* self (Xu et al., 2019).

These four previous *Tai-Chi* models draw heavily on the core concepts of *Yang* and *Yin*. *Yin* and *Yang* are opposite forces in the universe, but paradoxically they also are complementary, inter-connected, and interdependent. Each gives rise to the other as it interacts with it (Liu et al., 2016; Wang et al., 2019). According to the *I-Ching*, the distinction between *Yin* and *Yang*, as is true for other such dichotomies, is not real. The duality of *Yin* and *Yang*, as well as the opposite selves they represent, are an indivisible whole (Jung, 1973; Anagarika, 1981). According to the *I-Ching*, the core wisdom of Heaven’s rule is that the one generates two forms, then the two forms generate four images, and the four images generate the eight trigrams. The eight trigrams are symbolic representations of eight phenomena in nature (Xu et al., 2019). The 64 types of situations encountered by the self are represented by the 64 pairwise permutations of the eight trigrams, called the hexagram. This core wisdom is a kind of holistic knowledge of the formation and operation of all things in the universe (Anagarika, 1981). However, the four models all share a common weakness. These *Yang* and *Ying* concepts together form what has been calledcalled a “root metaphor” (Anagarika, 1981; Liu, 2013), which can be used to represent many dichotomies such as heaven/earth, male/female, day/night, and bright/dark. Such an ambiguous construct as root metaphor can hardly be used to develop a scientific theory (Hwang, 2013). Moreover, the four models fail to explain how the concept of *Tai-Chi* evolved over time or to provide a systematic explanation of how a comprehensive *I-Ching* model works.

Accordingly, there is a gap between the *I-Ching* cultural system and psychology. To fill in this gap, our epistemological strategy was to use a formal model of the self to analyze Confucian commentaries on the *I-Ching* with a view to constructing a culture-inclusive theory of psychology. This strategy required us to pay more attention to the internal mechanism of the *I-Ching* while heavily drawing on accounts of the self-cultivation process for becoming a *jun-zi*.

## The present work: *Jun-zi* self-cultivation model

The rest of article consists of four sections. The first section introduces the evolution of the *I-Ching* in Chinese history. In the second section, we describe the formal Mandela Model of Self (MMS), which we used to analyze the concept of *jun-zi*. In the third section, we present the JSM and compare various aspects of the psychological functioning of egoism with that of the *jun-zi* self and describe their respective underlying processes. Finally, potential theoretical directions, clinical applications, and suggestions for further research are provided in the conclusion section.

### The *I-Ching* cultural system: A cultural evolution perspective

The *I-Ching* is a very comprehensive book of great scope that embraces everything in nature. It includes rules for Heaven, man, and earth. (易之為書也, 廣大悉備, 有天道焉, 有人道焉, 有地道。) (*I-Ching*, The Great Appendix II, Chapter 70). The original version of the *I-Ching*, given the name Fu-Xi Yi (伏羲易), developed the first eight trigrams to explain the eight principles governing the natural world (Wilhelm et al., 1967; Anagarika, 1981). The subsequent king of Zhou, King-Wen Yi (文王易) developed the second version of *I-Ching*, also called Zhou Yi (周易), establishing the last eight diagrams to explain the ethical principles governing social interactions in daily life.

The *Yi-Wei* is an annotated supplement to the *I-Ching* written anonymously during the Spring and Autumn period (770–476 B.C.) and Warring states (475–221 B.C.) periods to predict good and bad fortune (Lin, 2002). In the Han Dynasty, the interpretations of the *I-Ching* were divided into two schools. Taoism and the *Yin-Yang* School used the *Yi-Wei* (易緯) to interpret the “image-numbers” (象數, *xiang-shu*) of the *I-Ching*, and thus the *Yin-Yang* School became the Image-Numbers School (象數派) (Hwang, 2023). However, Confucianists used the *I-Ching* to explain the ethical principles governing social interactions in daily life, and thus Confucianism came to be called the Philosophical School (義理派) (Hwang, 2023). But the Image-Numbers School was rejected by Confucian scholars during the Han Dynasty, because it merged with the *Chen-Shu* (讖書), whose members practiced a controversial form of divination called *Chen-Wei* (讖緯).

At the end of the Five Dynasties period (907–960 A.D.), the Taoist master Chen Tuan (陳摶) wrote “The Dragon’s Metaphor of the *I-Ching*” (龍圖易, *Long-Tu Yi*) which integrated the hexagrams of Fu-Xi and King Wen of Zhou with the five elements (五行, *wu-xing*) (metal, wood, water, fire, and earth) of *Yin* and *Yang*, which compose everything in the universe. By this time, the *I-Ching* had become a complete cultural system (Hwang, 2023). Later, Zhu Xi (朱熹) of the South Song Dynasty transformed the pre-Qin Confucianism of Dao-Xue (道學) into “Neo-Confucianism” (理學, *Li-Xue*), which served as a cosmology of the *I-Ching*.

The Confucian scholars who subscribe to *Li-Xue* (理學) emphasize the importance of practicing self-cultivation by diligently adhering to the rules of Heaven (天理). This means that all people should follow the rules of Heaven to find their own heavenly nature (天地之性) by overcoming egoistic desires (存天理, 滅人欲, *cun-tian-li*, *mie-ren-yu*). This guided the development of the JSM, described below.

### The mandala model of self

The MMS can be considered as a formal and universal model that describes the functioning of self striving for psychosocial equilibrium in every culture (Hwang, 2011; Shiah and Hwang, 2019). According to this model, people living in their lifeworld are symbolized by a circle surrounded by a square. Jaffe (1964) noted that the circle represents the ultimate wholeness or well-functioning of self, while the square symbolizes secularism, the flesh, and objective reality in a variety of cultural contexts. Therefore, the mandala can be regarded as a symbol for the prototype or deep structure of self in different cultures. It allows us to construct any culture specific model of self that illustrates the relationships between an individual’s attributes, actions, and cultural heritages, so as to contribute to the development of indigenous psychology (Hwang, 2011, 2019).

#### Four aspects of self: Individual, wisdom, action, and person

In the MMS, the self is being influenced by forces from four aspects, namely, individual, wisdom, action, and person. The psychological aspect of self is the locus of empirical experience in daily life. It takes various actions in the social context, and may engage in self-reflection when blocked from attaining its goals. In the conceptual framework of, all four of these terms are located outside the circle but within the square. This arrangement of terms means that one’s self is being influenced by forces from the individual’s external environment (Hwang, 2011). Each of the four forces has a distinct implication for mental health, as we will discuss below.

Anthropologist Harris (1989) proposed that, as the universal structure of personality, the terms “person,” “self,” and “individual” have different meanings in Western academic tradition. The individual is usually conceived as desire-driven biological entity that gives the self a personal identity or uniqueness, a feeling of ownership of various phenomena in the mind, body, and external world. This aspect of self is the main source of emotional disturbance for it follows hedonic principle with a deep-seated, reflexive false belief (Shiah, 2016). Paradoxically, the emotional disturbance caused by an individual’s desires provides us a good window for cultivating the self.

The person is cultural and sociological aspect of one’s personality; it is conceptualized as an agent-in-society that embodies actions in line with the appropriate and permitted behaviors to attain a specific goal for a certain role in the social order (Giddens, 1984, 1993). Every culture has its own definitions of appropriate and permitted behavior, each category of actions is endowed with a specific meaning and value that is transmitted to the individual through various channels of socialization.

According to Mandala Model of Self (Shiah and Hwang, 2019), the self as the subject of agency is endowed with two important capabilities: socialized reflexivity and knowledgeability. Knowledgeability is the ability of the self to memorize, store, and organize various forms of knowledge into a well-integrated system that guides reflexivity and action. Self-reflexivity is a process that monitors and explains its actions. When individuals intend to act, their decisions may be influenced by all four forces. This dynamic model of self can be used as a tool for cultural system analysis.

### *Jun-zi* self-cultivation model

#### The mandela model of self of *Jun-zi*

**FIGURE 1:**
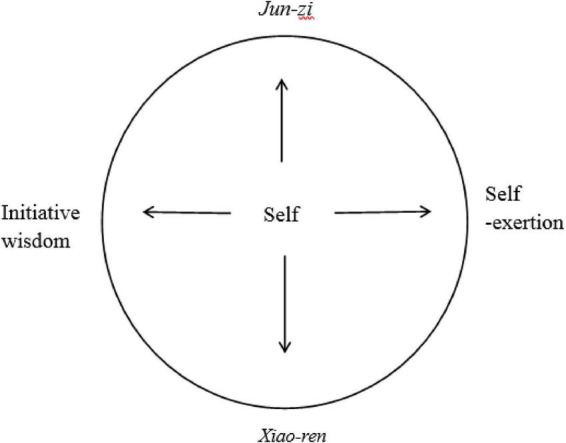
The mandela model of self (MMS) of *Jun-zi*.

As mentioned in earlier section, the MMS has been influenced by forces from four aspects of the self: individual, wisdom, action, and person. According to the MMS (Figure 1), a true gentleman (*jun-zi*) is an ideal person with the initiative wisdom to practice self-exertion (Figure 1), whereas a *xiao-ren* (小人) is an individual who follows the hedonic principle of pursuing stimulus-driven pleasure.

The wisdom of *jun-zi* can be seen in the following judgment of an *I-Ching* hexagram: “The vigorous Heaven is the initiative source and it provides all things with the vitality to grow. It is also the authority that governs everything. The wisdom of ‘self-exertion’ is to achieve the goal of being a *jun-zi* by actively initiating behavior based on Heaven’s rules and exert great effort to overcome difficulties.” But the individual has an instinctual tendency to do the opposite, following the principles of the self, such as egocentrism, self-centeredness, and biased self-interest.

According to the MMS of *jun-zi* (Figure 1), to become an ideal “person” (*jun-zi*) one has to follow the rules of Heaven (天理) in dealing with every situation that arises in daily life. On the other hand, if one takes into account only the interests of the “individual” as such and tries every means to meet the individual’s desires, then one might be considered a *xiao-ren*.

#### *Jun-zi* and egoism (*xiao-ren*): A comparison of their psychological functioning

**FIGURE 2:**
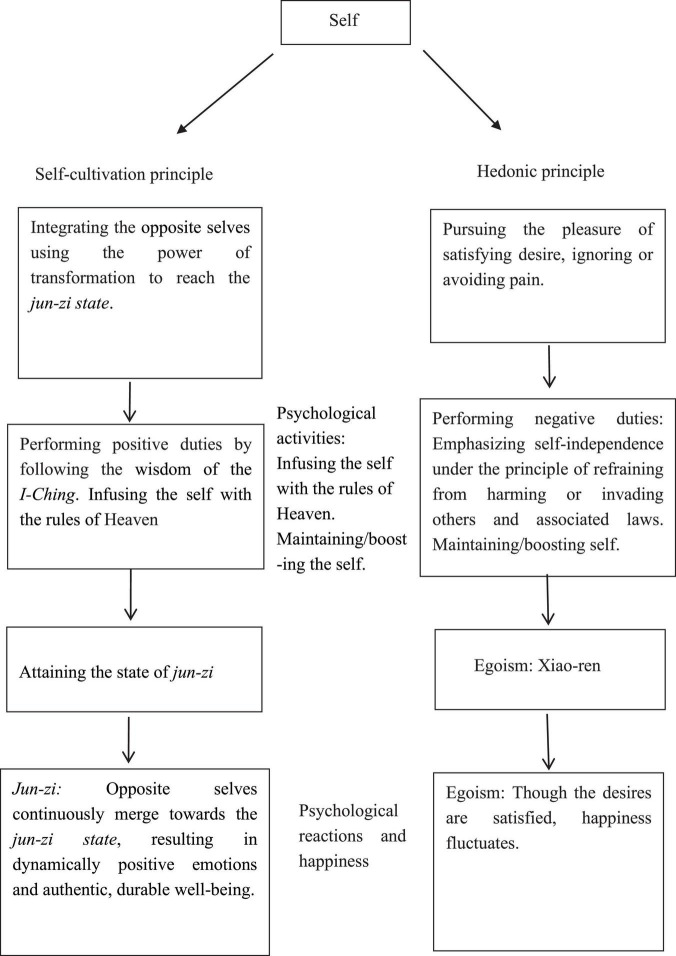
A comparison between the psychological functioning of *jun-zi* and *xiao-ren*.

Based on *I-Ching* teachings and Taoism, this section will elucidate the psychological functioning of two different types of self: the egoistic self and the *jun-zi* (Figure 2). Individuals of egoism follow the principle of seeking greater happiness and avoiding pain (Fave et al., 2011; Crespo and Mesurado, 2015). By adopting the negative duties of refraining from harming or invading others, and the associated laws, their psychological activities signify egoism mostly (Dambrun and Ricard, 2011; Shiah, 2016; Dambrun et al., 2019; Kuo et al., 2022).

In contrast, the self of a *jun-zi* applies self-cultivation by following the principle of integrating the opposite selves, thus creating a transformative power for the appropriate result. The psychological activities of a *jun-zi* consist of adopting the positive duties by obeying Taoist or Confucian wisdom to overcome the delusion of egoism (Xu et al., 2019; Yang, 2019). The goal of the self-cultivation process is to integrate, internalize, and simulate the operating rules of Heaven by striving for the meaningful experience of a dynamic balance between opposite selves, culminating in total harmony. This realization does not require one to deny the reality of every feature ascribed to the self, a move that some would find implausible. The word “harmony” should be defined as absence of the sense of those delusional features of the opposite selves that we ascribe to a single self, so it can contribute to the destruction of self-delusion (Xu et al., 2019; Hwang, 2023).

In the context of *I-Ching* wisdom, an individual, or *xiao-ren*, can become a true gentleman, or a *jun-zi*, who by practicing self-cultivation can attain good fortune and avoid disasters. *Jun-zi* persons, guided by wisdom from the *I-Ching*, exert great effort to overcome life’s difficulties. They do everything they need to do to promote good health and deal with every situation that may arise in a pro-active manner and with a modest demeanor. Practicing these virtues allows the *jun-zi to* accept every challenge with equanimity and provides the ability to maintain good mental and physical health throughout life. On the contrary, *xiao-ren* individuals mainly pursue the pleasure of satisfying their desires while trying to ignore or avoid the pain that always accompanies this pursuit, inevitably leading to unpleasant or even disastrous results.

Finally, the opposite selves are continuously integrating and merging toward the state of jun-zi; its achievement results in dynamically positive emotion and authentic, durable wellbeing. On the contrast, individuals who apply egoism can pursue the satisfaction of desires by using the hedonic approach. Though the desires are satisfied temporarily, happiness fluctuates. The criticism of egocentric happiness has been supported by recent research demonstrating that the hedonic principle does not lead to lasting happiness (Crespo and Mesurado, 2015; Dambrun et al., 2019; Juneau et al., 2020).

## Conclusion

To fill the gap left by the four previous *Tai-Chi* models, we present in this article the *jun-zi* Model of Self (JMS) based on teachings from the *I-Ching* and *Daodejing*. In recent decades, an increasing number of psychotherapists, counselors, and mental health workers began to pay attention to the value of traditional oriental classic texts about psychological healing (Cleary, 1993; Hwang, 2009; Wong and Liu, 2018). For instance, Chinese Taoist Cognitive Psychotherapy (CTCP) (Zhang et al., 2002) and Taoist Psychotherapy (Hwang, 2009; Shiah, 2021) adopt Taoist wisdom for instruction in psychotherapy. Although the JSM framework proposed in the present paper is still in its infancy, it was constructed on the very robust foundation of Taoist teachings and practices that have been applied for more than 4,800 years. Thus, Taoist practices may help people to reach their full human potential by accommodating their suffering and psychological illnesses to daily life. The JSM is a comprehensive model for self-cultivation that suggests many avenues for further investigation. Firstly, it offers a new perspective on humanity that differs from the Western conceptualization of personality, which emphasizes the satisfaction of the individual’s desires. Secondly, future research is needed to develop a psychometrically sound scale to measure the JSM and to explore the role of the JSM in the promotion of mental health. Lastly, the JSM was developed in a stepwise fashion.

More studies are needed to support this approach so as to elucidate the related social behavior. We hope that ordinary people will employ the JSM to improve their long-term mental health and wellbeing.

## Data availability statement

The original contributions presented in this study are included in the article/supplementary material, further inquiries can be directed to the corresponding author.

## Author contributions

JX and Y-JS wrote the first draft. N-SC and Y-FH contributed to writing the manuscript. All authors have read and approved the final manuscript.
